# Necrotizing pancreatitis complicated by retroperitoneal emphysema: two case reports

**DOI:** 10.1186/s40792-022-01542-2

**Published:** 2022-09-27

**Authors:** Kohei Chida, Keinosuke Ishido, Yoshiyuki Sakamoto, Norihisa Kimura, Hajime Morohashi, Takuya Miura, Taiichi Wakiya, Hiroshi Yokoyama, Hayato Nagase, Daichi Ichinohe, Akiko Suto, Daisuke Kuwata, Aika Ichisawa, Akie Nakamura, Daiki Kasai, Kenichi Hakamada

**Affiliations:** Department of Gastroenterological Surgery, Hirosaki University Graduate School of Medicine, Hirosaki, Japan

**Keywords:** Pancreatitis, Necrosis, Emphysema, Gas

## Abstract

**Background:**

Emphysematous pancreatitis is acute pancreatitis associated with emphysema based on imaging studies and has been considered a subtype of necrotizing pancreatitis. Although some recent studies have reported the successful use of conservative treatment, it is still considered a serious condition. Computed tomography (CT) scan is useful in identifying emphysema associated with acute pancreatitis; however, whether the presence of emphysema correlates with the severity of pancreatitis remains controversial. In this study, we managed two cases of severe acute pancreatitis complicated with retroperitoneal emphysema successfully by treatment with lavage and drainage.

**Case presentation:**

Case 1: A 76-year-old man was referred to our hospital after being diagnosed with acute pancreatitis. At post-admission, his abdominal symptoms worsened, and a repeat CT scan revealed increased retroperitoneal gas. Due to the high risk for gastrointestinal tract perforation, emergent laparotomy was performed. Fat necrosis was observed on the anterior surface of the pancreas, and a diagnosis of acute necrotizing pancreatitis with retroperitoneal emphysema was made. Thus, retroperitoneal drainage was performed. Case 2: A 50-year-old woman developed anaphylactic shock during the induction of general anesthesia for lumbar spine surgery, and peritoneal irritation symptoms and hypotension occurred on the same day. Contrast-enhanced CT scan showed necrotic changes in the pancreatic body and emphysema surrounding the pancreas. Therefore, she was diagnosed with acute necrotizing pancreatitis with retroperitoneal emphysema, and retroperitoneal cavity lavage and drainage were performed. In the second case, the intraperitoneal abscess occurred postoperatively, requiring time for drainage treatment. Both patients showed no significant postoperative course problems and were discharged on postoperative days 18 and 108, respectively.

**Conclusion:**

Acute pancreatitis with emphysema from the acute phase highly indicates severe necrotizing pancreatitis. Surgical drainage should be chosen without hesitation in necrotizing pancreatitis with emphysema from early onset.

## Background

Emphysematous pancreatitis is a severe form of acute pancreatitis associated with emphysema in imaging studies and has been considered a subtype of necrotizing pancreatitis [1]. In this report, we describe two patients with severe acute necrotizing pancreatitis complicated with retroperitoneal emphysema relieved by emergency surgery.

## Case presentation

### Case 1

**Fig. 1:**
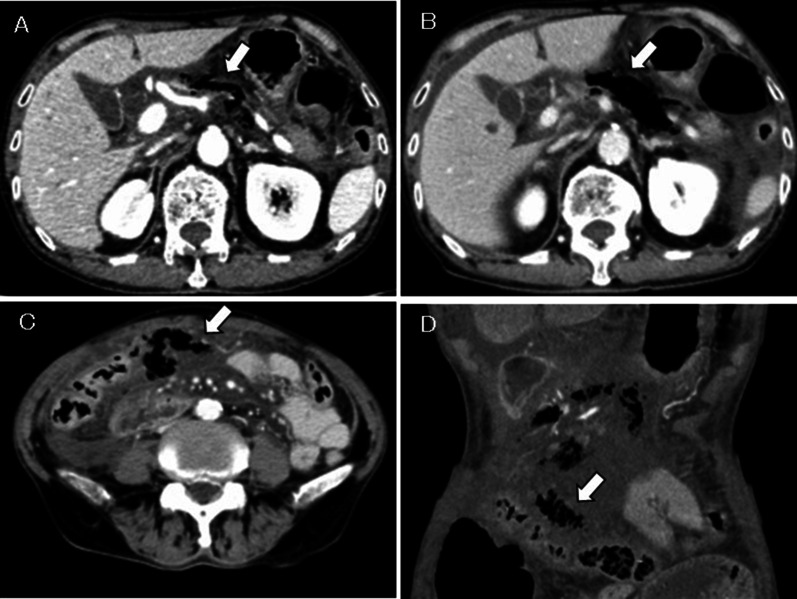
Contrast-enhanced CT scan of case 1. **A** Initial CT showing the emphysema around the pancreatic body (arrow). **B** CT performed 6 h post-admission showing a significant increase in retroperitoneal gas (arrow). **C**, **D** CT performed 6 h post-admission showing the appearance of gas in the transverse mesocolon (arrows)

A 76-year-old man visited his previous doctor due to vomiting after consuming lunch. Computed tomography (CT) scan showed signs of pancreatitis. He was referred to our hospital the next day. His medical history included untreated diabetes mellitus and gastric ulcer, and he also had a history of alcohol consumption of approximately 60 g per day. On arrival, his pulse, blood pressure, and body temperature were 120 beats/min, 109/68 mmHg, and 37.5℃, respectively. Abdominal findings included mild tenderness in the upper abdomen without peritoneal irritation signs. Laboratory findings on admission were white blood cell (WBC) of 2060 /µL; hemoglobin (Hb), 16.2 g/dL; platelet count (Plt), 16.9 /µL; aspartate transaminase, 48 U/L; alanine transaminase (ALT), 18 U/L; creatinine (Cre), 0.8 mg/dL; blood urea nitrogen (BUN), 21 mg/dL; lactate dehydrogenase (LDH), 254 U/L; amylase (AMY), 1023 U/L; glucose level, 253 mg/dL; glycated hemoglobin 7.3%; and C-reactive protein (CRP), 4.48 mg/dL. Abdominal contrast-enhanced CT scan showed extensive emphysema in the retroperitoneum, increased adipose tissue density around the pancreatic body, and ascites around the pancreatic body (Fig. 1A).

**Fig. 2:**
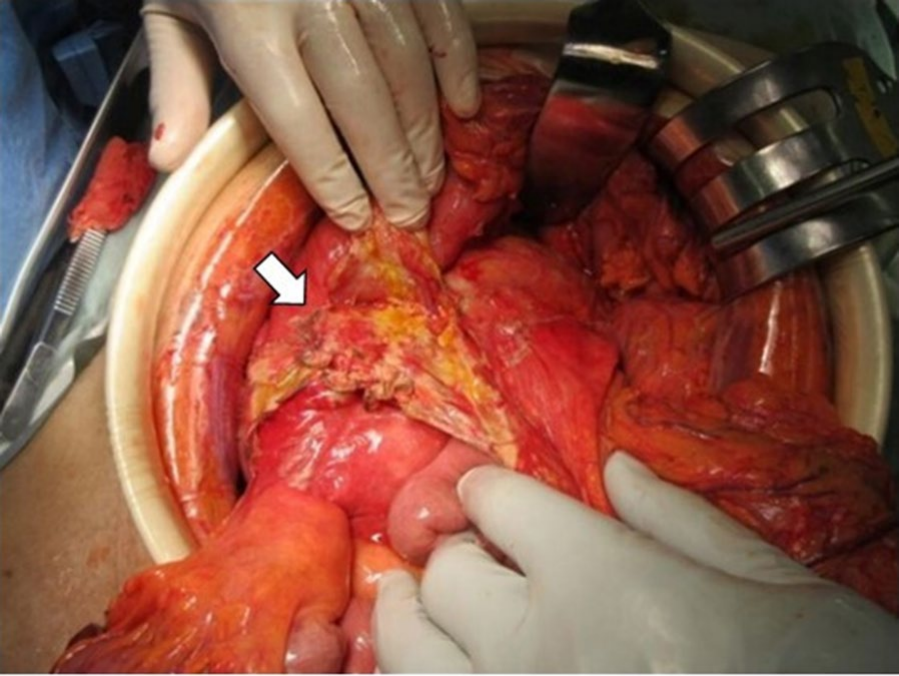
Surgical view of case 1 showing the necrotic change of pancreas and peripancreatic tissue (arrow)

Based on the above data, conservative treatment was initiated based on acute pancreatitis and peripancreatic abscess diagnoses. However, immediately after initiating the treatment, peritoneal irritation symptoms occurred. A CT scan was performed again 6 h post-admission and showed no evidence of free gas in the abdominal cavity; however, a significant increase in retroperitoneal gas (Fig. 1B) and the appearance of gas in the transverse mesocolon was observed (Fig. 1C, D). Therefore, with the suspicion of gastrointestinal tract perforation, an emergency laparotomy was performed. During laparotomy, a moderate amount of clear yellow ascites fluid was detected, and edematous changes and inflammatory wall thickening were observed in the transverse colon. Necrotic changes of the pancreatic parenchyma were observed in the anterior pancreatic body, with retroperitoneal emphysema in the dorsal pancreatic body (Fig. 2). When the retroperitoneum was opened, air and purulent effusion were observed. Considering the possibility of necrotizing pancreatitis and transverse colon perforation, partial transverse colon resection, colostomy, lavage, and abdominal drainage were performed. Abscess formation was observed within the removed transverse colon mesentery; however, no grossly visible perforation was observed in the transverse colon. *Klebsiella pneumoniae* was detected in the purulent fluid collected at the time of surgery, which was consistent with blood culture results at the time of the initial examination. The patient had a good postoperative course, and thus, his pancreatitis was quickly resolved. The patient had no postoperative infectious complications and was discharged on the 18th postoperative day. Eight months after discharge from the hospital, a colostomy closure was performed after confirming his good postoperative recovery.

### Case 2

**Fig. 3:**
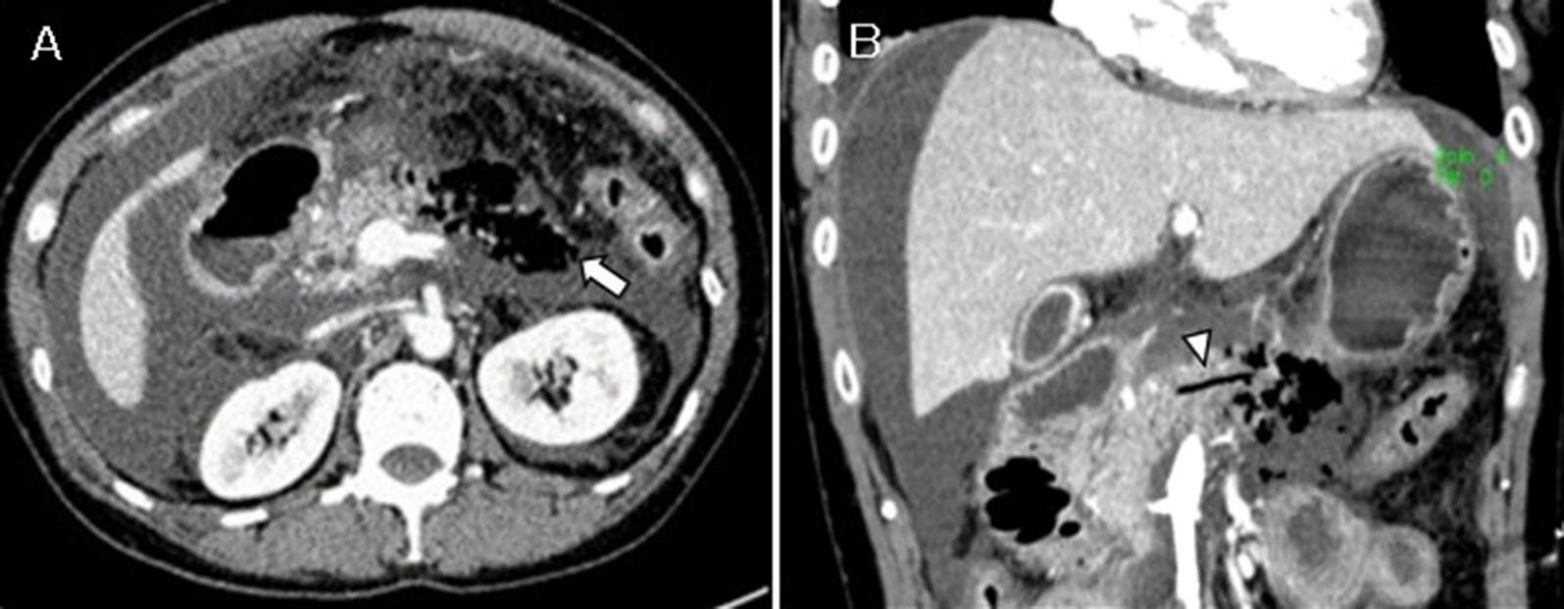
Contrast-enhanced CT of case 2. **A** It shows the emphysema around the pancreatic body including the retroperitoneal space (arrow). **B** Coronal view of the same CT scan showing emphysema in the pancreatic duct (arrowhead)

A 50-year-old woman developed anaphylactic shock during the induction of general anesthesia for a planned surgery for a lumbar disk herniation at a previous hospital, and surgery was canceled. On the same day, severe abdominal pain occurred, and she was transferred to our hospital the next day because of the suspected acute pancreatitis on a contrast-enhanced CT scan. Upon arrival, her pulse, blood pressure, and body temperature were 120 beats/min, 90–100 mmHg with noradrenaline of 0.25γ, and 39.1 °C, respectively. She was drowsy, however, and complained of abdominal pain. The abdomen was generally distended with involuntary defense. Livedo reticularis was also observed on the extremities. Her only previous medical history was a lumbar disk herniation, and her social history was unremarkable. On admission, laboratory findings were WBC of 1320 /μL; Hb, 17.3 g/dL; Plt, 253,000 /μL; AST, 97 U/L; ALT, 42 U/L; Cre, 1.59 mg/dL; BUN, 18 mg/dL; LDH, 389 U/L; AMY, 1629 U/L; and CRP, 19.36 mg/dL. An abdominal contrast-enhanced CT scan showed a partial area of necrosis in the pancreatic body with extensive emphysema in the surrounding retroperitoneal cavity. Although no intra-abdominal free gas was observed, a large amount of ascites was observed (Fig. 3). Based on these data, the patient was diagnosed with necrotizing pancreatitis with emphysema and septic shock, and an emergency laparotomy was performed.

**Fig. 4:**
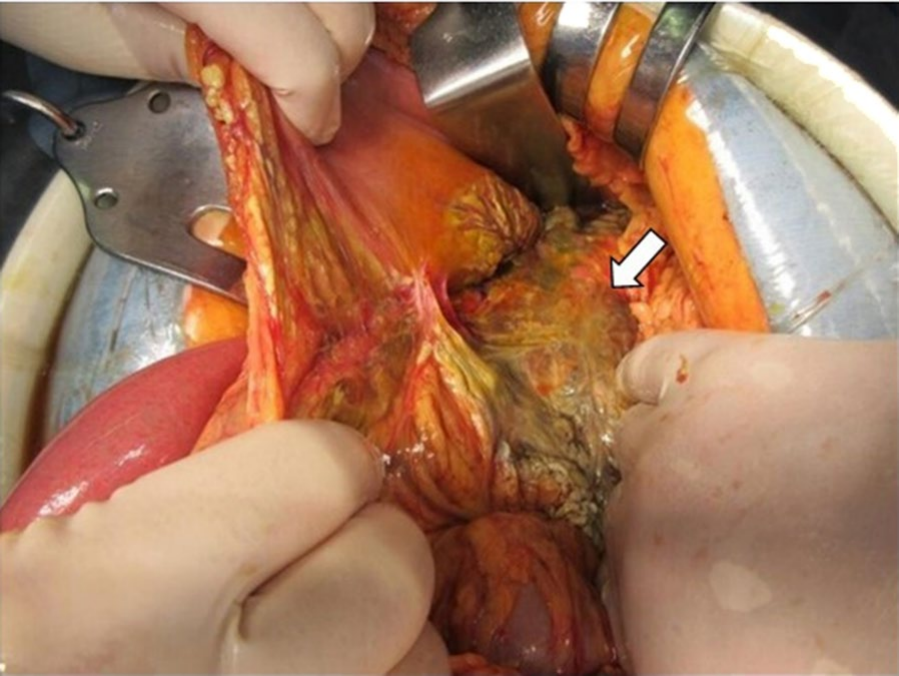
Surgical view of case 2 showing extensive necrotic change of pancreas (arrow) and emphysema around the pancreatic body including the retroperitoneum

Intraoperatively, a large amount of turbid ascites fluid was observed. When the omental foramen was opened, necrotic changes were observed in the pancreatic body and surrounding fatty tissues (Fig. 4). No gastrointestinal perforation was observed. The retroperitoneum around the pancreatic body was extensively opened, and fatty tissues with necrotic changes were removed as much as possible. Although necrotic changes were observed in the pancreatic body, the pancreatic parenchyma was preserved, and resection was not necessary. Therefore, lavage and drainage were performed, and drains were placed in the abdominal and retroperitoneal cavities. The purulent ascites fluid collected at the time of surgery was found to contain *Escherichia coli*, which was consistent with blood culture results during the initial examination.

The patient had a good postoperative course, and vasopressors were discontinued on the 2nd postoperative day. The patient was transferred to the ward from the intensive care unit on the 8th postoperative day. However, the patient developed an organ-space surgical site infection around the pancreatic body. The site infection was refractory, requiring several image-guided drain replacements. Following adequate drainage treatment, the drainage tube was removed on the 101st postoperative day. Consequently, the patient was discharged on the 108th postoperative day.

## Discussion

We report two patients with acute necrotizing pancreatitis with retroperitoneal emphysema, which developed rapidly and was associated with intra-abdominal infection requiring emergency laparotomy drainage. Both patients were successfully treated with abdominal drainage and improved from a severe condition, and the pancreatitis was treated conservatively.

Emphysematous pancreatitis is a subtype of necrotizing pancreatitis and is considered as acute pancreatitis with emphysema in and around the pancreas [1]. It has been suggested to be associated with alcohol consumption, diabetes mellitus, history of abdominal surgery, history of pancreatitis, and gallstones [2]. A history of alcohol consumption of approximately 60 g/day and untreated diabetes mellitus may have been associated with the onset of the disease in case 1. In case 2, the patient developed severe anaphylactic shock during induction of anesthesia for lumbar hernia surgery, rapidly followed by necrotizing pancreatitis, suggesting that drug-induced pancreatitis during induction of anesthesia or pancreatic ischemia due to a sudden drop in blood pressure in the anaphylactic reaction may have been associated with the occurrence of necrotizing pancreatitis. In general, muscle relaxants and antimicrobial agents are the most frequently suspected drugs that result in allergic reactions during induction of anesthesia [3]. Furthermore, several causes of pancreatic tissue ischemia such as vasculitis, atheroembolism, intraoperative hypotension, and hemorrhagic shock were reported [4–8]. In one report, 81 of 300 patients (27%) who underwent cardiac surgery developed hyperamylasemia, and three patients subsequently developed necrotizing pancreatitis [7]. Thus, pancreatic tissue ischemia due to anaphylactic shock may have triggered the occurrence of necrotizing pancreatitis. On the other hand, propofol is also reported as a cause of pancreatitis [9]. Therefore, these drugs in the induction of anesthesia may have contributed to the onset of pancreatitis in our case 2. Reports of emphysematous pancreatitis by Bul et al*.* revealed that the majority of isolated bacteria were Gram-negative enterobacteria [2]. Conversely, about half of the patients were Gram-positive organisms on culture, most of which were *Clostridium perfringens* [2]. These bacteria ferment glucose in the necrotic tissues, producing carbon dioxide and nitrogen gas [10]. Patients with poorly controlled diabetes more likely develop infections with emphysema due to increased tissue glucose concentration in the interstitial fluid caused by impaired glycolytic function [10]. However, the pathways by which these bacteria spread to the pancreas and surrounding tissues at the onset of the necrotizing pancreatitis are unclear; hematogenous and lymphatic migration from the gastrointestinal lumen to the peripancreatic area, backflow from the Vater’s papilla into the bile or pancreatic duct, or through a gastrointestinal tract fistula [2]. In our both cases, Gram-negative rods were detected in the peripancreatic abscess and venous blood, *Klebsiella pneumoniae* in one case, and *Escherichia coli* in the other case. No fistula was observed between the peripancreatic abscess and gastrointestinal tract, and the environment was not associated with biliary infection, suggesting the involvement of bacterial translocation. Any shock that disrupts the normal intestinal barrier can compromise the barrier function, resulting in bacterial translocation and infection in humans [11]. Bacterial translocation in humans is assumed to occur in several clinical manifestations—bacterial overgrowth in the small intestine, intestinal barrier damage (secondary changes in the intestinal microvasculature due to shock, systemic inflammatory response syndrome, and direct injury), systemic immunosuppressed status [12, 13], hemorrhagic shock [14], acute pancreatitis, cirrhosis, obstructive jaundice, and abdominal surgery [15, 16]. In our cases, bacterial translocation associated with acute pancreatitis may have caused intestinal bacteria proliferation in ischemic and necrotic pancreatic tissues, which could have further amplified the inflammation.

Up to 10%–20% of patients with acute pancreatitis are associated with pancreatic necrosis, surrounding pancreatic tissue necrosis, or both, and when infection occurs in the necrotic tissue, the mortality rate reaches 20% to 30% [17]. In the past, open drainage was the first choice of treatment for emphysematous pancreatitis; however, in recent years, there have been cases of successful conservative treatment [18, 19], and percutaneous or endoscopic drainage has been reportedly associated with lower mortality than open drainage [20–22]. In general, infectious complications of pancreatitis generally occur > 2–3 weeks after the initial treatment [23]. Furthermore, since early surgery for severe necrotizing pancreatitis is associated with a worse prognosis, endoscopic or percutaneous drainage has been reportedly performed at 4 weeks post-onset of pancreatitis if the patient’s general condition is maintained with conservative treatment [17]. In other words, percutaneous or endoscopic local infection drainage after acute pancreatitis should be performed, except in the following situations—failure of conservative treatment or medical drainage, undeniable perforation of the gastrointestinal tract as a cause of emphysema, and complications of abdominal compartment syndrome. Further, Beger et al*.* reported a 24% bacterial contamination rate of necrotic material in patients operated on during the first 7 days after the onset of acute necrotizing pancreatitis, suggesting that infection may be complicated early during pancreatitis [24]. In the general case of severe necrotizing pancreatitis, most patients have a severe course due to systemic inflammation associated with pancreatitis even after performing open surgical drainage. However, the retroperitoneal infection that was concomitant with acute pancreatitis was thought to greatly contribute to the disease severity in our cases. Therefore, early surgical drainage may have led to infection control. Hence, drainage procedures aimed at early removal of infected tissues may be effective even in pancreatitis.

A Pubmed search for the keywords “Emphysematous”, “Pancreatitis”, “Gas”, and “Air” over a 20-year period from 1992 to 2021 identified 58 reports of acute pancreatitis with emphysema, which were reviewed in 60 patients, including these two patients (Table 1). The median age was 61 years, comprising 46 males, 12 females, and 2 of unknown sex. The prevalence of diabetes and history of alcohol consumption was lower than in previous reports; age could be a risk factor, since the median age in the analysis of 60 cases was 61. In our review, *Escherichia coli* and *Klebsiella pneumoniae* were most frequently detected bacteria in the peripancreatic area, but the clinical course did not differ depending on the organisms detected. There were 12 cases in which positive blood cultures were observed; however, the exact rate of positive blood cultures was unknown since most of the previous reports did not indicate it. A total of 20 patients underwent laparotomy, 13 underwent percutaneous or endoscopic drainage therapy, 4 patients underwent both laparotomy and drainage therapy, and 22 underwent conservative treatment. Then, 17 of 60 patients died, for an overall mortality rate of 27%, similar to previous reports on mortality rates caused by necrotizing pancreatitis [66]. However, 34 (56.7%) patients had < 1 week between the onset of pancreatitis and the occurrence of emphysema, and 15 of these patients died. The mortality rate was as high as 44.1%, and the early occurrence of emphysema was a particularly serious condition. Most of the 15 patients who developed emphysema within a week of disease onset died from multiple organ failures. Thus, the early onset of acute pancreatitis with emphysema may lead to rapid disease progression. Although the causes of retroperitoneal emphysema range from severe to benign diseases, infection associated with emphysema may be rapidly progressing to necrotizing infection, as in gas gangrene. Moreover, in these clinical situations, conservative treatment and percutaneous or endoscopic drainage may be preceded by surgical treatment; however, we should not hesitate to resort to surgical treatment to ensure adequate drainage.

**Table 1:** A summary of necrotizing pancreatitis with emphysema

60 cases including our two cases and 58 reported cases (Ref. [2, 18–20, 25–65])	
Sex	
Male	46 (76.7%)
Female	12 (20.0%)
Unknown	2 (3.3%)
Age, median [IQR]	61 [51.5–73]
Co-morbid disease	
History of diabetes mellitus	17 (28.3%)
History of drinking	
Yes	20 (33.3%)
None	40 (66.7%)
Days to appearance of emphysema from disease onset	
< 7 days	34 (56.7%)
≥ 7 days	15 (25.0%)
Unknown	11 (18.3%)
Bacteria detected in the peripancreatic area	Number of cases
Total	38
*E. coli*	14
*K. pneumoniae*	9
*C. perfringens*	7
*P. aeruginosa*	3
*Enterococcus* spp.	2
*E. aerogenes*	2
*Candida* spp.	2
*P. vulgaris*	1
*H. parainfluenzae*	1
*S. sanguis*	1
*Bacteroides* spp.	1
Not mentioned	22
Treatment	
Laparotomy	20 (33.3%)
Puncture drainage (endoscopic or percutaneous)	13 (21.7%)
Combined (laparotomy and puncture drainage)	4 (6.7%)
Conservative treatment	23 (38.3%)
Prognosis	
Alive	43 (71.7%)
Dead	17 (28.3%)
< 7 days to appearance of emphysema from disease onset	15
≥ 7 days to appearance of emphysema from disease onset	0
Unknown	2
Mortality rate	
All patients	27%
< 7 days to appearance of emphysema from disease onset	44.1%

## Conclusions

Necrotizing pancreatitis complicated by emphysema is an extremely severe acute pancreatitis. In this study, two patients had severe necrotizing pancreatitis with retroperitoneal emphysema. In both patients, peripancreatic drainage including retroperitoneal space was successful. When emphysema is observed early in acute pancreatitis, the disease may progress rapidly; therefore, surgical treatment such as open drainage should be selected.

## Data Availability

Data will be made available by the corresponding author upon reasonable request.

## Abbreviation

CT: Computed tomography
